# Contextual determinants influencing the implementation of fall prevention in the community: a scoping review

**DOI:** 10.3389/frhs.2023.1138517

**Published:** 2023-05-11

**Authors:** M. C. van Scherpenseel, S. J. te Velde, C. Veenhof, M. H. Emmelot-Vonk, J. A. Barten

**Affiliations:** ^1^ Research Group Innovation of Human Movement Care, Research Center for Healthy and Sustainable Living, HU University of Applied Sciences Utrecht, Utrecht, Netherlands; ^2^ Department of Rehabilitation, Physiotherapy Science and Sport, University Medical Center Utrecht, Utrecht University, Utrecht, Netherlands; ^3^ Department of Geriatrics, University Medical Center Utrecht, Utrecht, Netherlands

**Keywords:** fall prevention, implementation, contextual determinants, community-dwelling older adults, scoping review

## Abstract

**Background:**

Successful implementation of multifactorial fall prevention interventions (FPIs) is essential to reduce increasing fall rates in community-dwelling older adults. However, implementation often fails due to the complex context of the community involving multiple stakeholders within and across settings, sectors, and organizations. As there is a need for a better understanding of the occurring context-related challenges, the current scoping review purposes to identify what contextual determinants (i.e., barriers and facilitators) influence the implementation of FPIs in the community.

**Methods:**

A scoping review was performed using the Arksey and O'Malley framework. First, electronic databases (Pubmed, CINAHL, SPORTDiscus, PsycINFO) were searched. Studies that identified contextual determinants that influence the implementation of FPIs in the community were included. Second, to both validate the findings from the literature and identify complementary determinants, health and social care professionals were consulted during consensus meetings (CMs) in four districts in the region of Utrecht, the Netherlands. Data were analyzed following a directed qualitative content analysis approach, according to the 39 constructs of the Consolidated Framework for Implementation Research.

**Results:**

Fourteen relevant studies were included and 35 health and social care professionals (such as general practitioners, practice nurses, and physical therapists) were consulted during four CMs. Directed qualitative content analysis of the included studies yielded determinants within 35 unique constructs operating as barriers and/or facilitators. The majority of the constructs (*n* = 21) were identified in both the studies and CMs, such as “networks and communications”, “formally appointed internal implementation leaders”, “available resources” and “patient needs and resources”. The other constructs (*n* = 14) were identified only in the studies.

**Discussion:**

Findings in this review show that a wide array of contextual determinants are essential in achieving successful implementation of FPIs in the community. However, some determinants are considered important to address, regardless of the context where the implementation occurs. Such as accounting for time constraints and financial limitations, and considering the needs of older adults. Also, broad cross-sector collaboration and coordination are required in multifactorial FPIs. Additional context analysis is always an essential part of implementation efforts, as contexts may differ greatly, requiring a locally tailored approach.

## 1. Introduction

Fall rates are expected to increase in the coming decades, due to the rapidly aging population and the rising prevalence of multimorbidity, polypharmacy, and frailty among older adults (1). Currently, more than one-third of community-dwelling people over the age of 65 years fall each year (2). Fall-related injuries may have significant personal consequences, such as short- and long-term functional impairment, reduction in quality of life, loss of independence and they may cause fractures, serious soft tissue injuries, and even death (3). In addition, falls in this population are the leading cause of emergency department visits and hospitalizations, which result in a high health care demand and, therefore, high fall-related health care costs (4, 5). As a result, reducing falls in community-dwelling older adults has become an international health priority (2, 4, 6).

In order to reduce fall rates, the use of multifactorial fall prevention interventions (FPIs) is recommended (7). Multifactorial FPIs are primarily designed to address known modifiable risk factors for falling, which have been identified through individual fall risk assessments (7, 8). These multifactorial FPIs consist, therefore, of different types and combinations of interventions, such as exercise therapy, medication review, and occupational therapy (3). This requires a multidisciplinary approach across individuals, providers, and organizations within the context where the FPIs occur (3). However, the potential of effective FPIs is often constrained due to a lack of successful implementation (6, 9). Failing to appropriately implement research findings into clinical practice severely limits the potential for patients, and communities as a whole, to benefit from advances of proven effective interventions.

To achieve successful implementation of a proven effective intervention into practice, implementation strategies must be applied (10, 11). Implementation strategies are methods or techniques used to improve adoption, implementation, and sustainability of a clinical practice or program (12). However, an implementation strategy may be effective in one setting and result in failure in another, since every organization, community, or provider experiences different barriers or facilitators during implementation depending on their context (13). Therefore, implementation strategies must be tailored to the unique, dynamic, local context where the implementation of the intervention occurs (10, 14). Tailoring strategies to specific contexts requires several steps, of which examining and understanding the local barriers and facilitators (i.e., contextual determinants) is the first one (15, 16). Within this step, the use of theoretical frameworks is highly recommended to better understand and explain which determinants account for the success or failure of a specific implementation strategy (17). Tools exist to help implementers to assess potential determinants in a specific context, such as the widely used Consolidated Framework for Implementation Research (CFIR) (17, 18).

Recently, McConville and Hooven (2020) (19) performed an integrative review to identify determinants that influence the implementation of fall prevention management in the primary care setting. Five themes were identified that described barriers to implementation: provider beliefs and practice, lack of provider knowledge, time constraints, patient engagement, and financial issues. However, this research mainly focused on barriers, whereas insight into facilitators is equally important for context analyses and future steps in the implementation process (10). Furthermore, they primarily concentrated on the perceptions of health care professionals, while responsibility for effective fall prevention management lies not with providers in health care, but also in social care sectors (3, 20). Nevertheless, many studies on the implementation of FPIs are still concentrating on single care settings or provider groups (21, 22).

Focusing on a single setting, organization or provider type has been long debated by Ganz et al. (2008) (23), where it was emphasized that “it takes a village” of stakeholders across settings, sectors, and organizations to prevent falls and reduce fall risk among older people. Concentrating on the implementation of FPIs in the community setting is therefore essential (3, 24). “Community setting” can be defined as the geographical area where community-based health and or social care services are delivered (integrally) to residents in primary or community care (25). Surprisingly, the role of communities as a context for FPIs has been mostly unrecognized. Understanding and accounting for what happens in the context of the community where the intervention is performed, is of major importance to better address implementation challenges (13). To date, little is known about the best ways to implement FPIs in the broader context of the community.

The first step to address this gap of knowledge is to gain insight in contextual determinants that influence the implementation process in the local context where the intervention is performed (16). Additionally, active involvement of relevant stakeholders is essential to add relevance and impact to findings derived from the literature. Therefore, a scoping review incorporating consultation with stakeholders was conducted, aiming to identify what contextual determinants influence the implementation of FPIs in the community.

## 2. Materials and methods

### 2.1. Design

This scoping review was conducted according to the methodological framework developed by Arksey & O'Malley (2005) (26) and further enhanced by the work of Levac et al. (2010) (27). There are two key stages to this methodology: (1) a comprehensive review of the literature; and (2) consulting stakeholders in the field during consensus meetings to inform or validate the findings from the literature. In the area of fall prevention in the community, health and social care professionals are key stakeholders. Also, it is suggested that researchers share preliminary findings as a foundation to inform the consultation and to enable stakeholders to build on the existing evidence (27).

Reporting was performed according to the Preferred Reporting Items for Systematic reviews and Meta-Analyses Extension for Scoping Reviews (PRISMA-ScR) checklist (Appendix 1) (28). This scoping review is part of a Dutch implementation research project: Fall pRevention ImplEmentatioN stuDy (FRIEND), which has received ethical clearance from the Ethical Committee Research Healthcare Domain of the HU University of Applied Sciences, Utrecht, the Netherlands (113-000-2020).

### 2.2. Identifying relevant studies

Studies in this review focus on contextual determinants influencing the implementation of FPIs from the perspective of health and social care professionals in the community. “Context” in this review is broadly defined as everything outside the evidence-based intervention and includes all forces (or “determinants”) working for or against implementation (18, 29). “FPI” is defined as a multifactorial evidence-based intervention in health and social care addressing modifiable fall risk factors and therefore aiming at fall prevention (such as exercise, medication review, occupational therapy, and nutrition therapy) (30).

Studies were eligible for inclusion in this review if they: (1) described barriers and/or facilitators regarding implementation of FPIs for community-dwelling older people; (2) were performed in a community setting; (3) had a (partly) qualitative study design; (4) were written in English or Dutch; and (5) were published since 2010. Only articles from approximately the last decade were included since this best reflects the current health care landscape. Furthermore, quantitative studies were not included since qualitative methods are suited best to discerning barriers and facilitators to the uptake of an intervention (31). Studies were excluded if they: (1) examined eHealth-interventions; (2) investigated the implementation of fall risk screening or assessment only; (3) focused solely on participants with mental health and/or neurological conditions (such as dementia or Parkinson's disease); (4) focused on perceptions of older people regarding FPIs only; (5) were intervention studies assessing the effectiveness of fall prevention interventions, such as Randomized Controlled Trials; or (6) were non-Western studies. The reason for excluding the latter was that the health systems of the Netherlands and other Western countries are more similar, in comparison with those of non-Western countries.

To identify potentially relevant studies, the following electronic databases were searched: Pubmed, CINAHL, PsychINFO, and SPORTDiscus. The search was supplemented by scanning the reference lists of included studies. The key search strategy consisted of the words “fall prevention intervention”, “barriers and/or facilitators”, “community”, and “implementation”. The search words were combined through Boolean operators. The search strategy was drafted by one researcher (MS) and further refined through discussion with another researcher (JB). The final search strategy was performed in March 2022 (Appendix 2). An update of the results derived from the initial search strategy was carried out in October 2022, to find the most recently published articles. In addition, initially, we explicitly left “RCTs” out of the search strategy to narrow down the results and to exclude effectiveness-studies. However, in order to ensure we did not miss any eligible studies that were documented as hybrid implementation trials involving randomization, the search strategy was rerun in March 2023 after deleting search terms related to the study design.

### 2.3. Study selection

Prior to study selection, agreement on selection criteria was reached to increase consistency among researchers. Then, the studies that arose from the search strategy were exported to Rayyan, a web app for reviews (32). Study selection comprised two stages. First, all titles and abstracts were screened independently by two reviewers (MS and JB). Second, if studies seemed to be eligible, the full text was reviewed independently (MS and JB). If disagreement on study selection arose, the researchers (MS and JB) discussed until they reached consensus. When conflicts were unresolved, a third researcher (SV) was approached. However, this proved to be unnecessary since consensus was reached (MS and JB) on eligibility after both stages. Finally, the reviewers generated a definitive list of studies eligible for inclusion.

### 2.4. Charting the data

A data-charting form was co-created by two researchers (MS and JB). Descriptive data of the included studies were extracted by one researcher (MS) in the data-charting form: authors, year of publication, country, study design, data collection, data analysis, type of FPI, setting, and study population. The findings were discussed with and confirmed by all members of the research team (MS, SV, CV, ME, JB).

### 2.5. Collating, summarizing and reporting results

**Figure 1:**
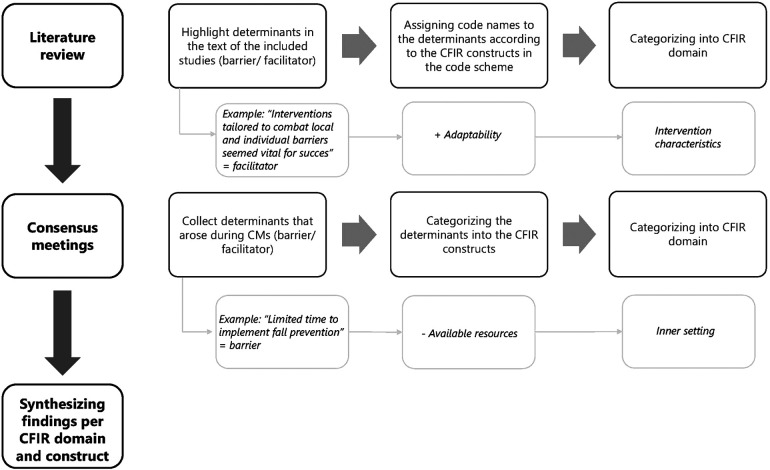
Process of analysis of the data from included studies during literature review, and consensus meetings within the current scoping review, with an example.

The data in the included studies were analyzed using directed qualitative content analysis (Figure 1) (33). Figure 1 shows the process of the data analysis, as well as examples of contextual determinants that derived from the literature and the CMs. Analysis was performed in ATLAS.ti, version 22®. Within this structured type of qualitative analysis, the first step is to identify key concepts or variables to create an initial coding scheme with predetermined codes (33). In this review, the constructs of the CFIR were used as predeterminant codes. The CFIR is among the most well-operationalized and widely used determinant frameworks to perform research within local settings (18). The original CFIR consists of 39 implementation constructs, categorized into five domains that influence implementation: *Intervention characteristics* (e.g., features and quality of the intervention), *Outer setting* (e.g., the economic, political, and social context), *Inner setting* (e.g., the structural, political and cultural context where the implementation takes place, such as an organization), *Characteristics of individuals* (e.g., attitudes, values and believes of the individuals involved) and *Process* (e.g., components that impact the implementation process) (18). Second, in the included studies, relevant determinants in the text were highlighted. Then, a differentiation was made between a determinant being a barrier (−), facilitator (+), or having no specific direction (+/−). Determinants were considered barriers if they hindered or impeded implementation; determinants were considered facilitators if their presence promoted implementation. Only when a determinant was explicitly mentioned to be a barrier or facilitator, it was coded as such. In all other cases, e.g., a determinant was “important to consider”, it was coded as +/−. A determinant might have been coded multiple times in the same study and with different allocations, e.g., as both a barrier (−) and as having no specific direction (+/−). This occurred when a determinant was specifically mentioned as being a barrier (−), and later on, was described without a specific direction (+/−). Third, the determinants were assigned to the CFIR constructs in the coding scheme and then categorized into the CFIR domains.

Of all studies that resulted from the search strategy, a quarter was independently coded by two researchers (MS and JB). After a consensus meeting, where differences were discussed until consensus was reached, one researcher coded (MS) the rest of the studies. Overall, the selected determinants in the text and appointed CFIR constructs were very similar between both researchers. Finally, findings were presented in a table according to the five domains of the CFIR and they were discussed with the research team, considering the meaning and overall implications of the results.

### 2.6. Consultation

To validate and complement the preliminary findings from the included studies to the context of Dutch communities and to offer an additional source of information, meaning, and perspective, stakeholders were approached to be included in consensus meetings (26, 27). A broad selection of health and social care professionals (HSCPs) working with fall prevention in four districts in the region of Utrecht, the Netherlands were involved in the FRIEND project, such as general practitioners, physical therapists, dieticians, community nurses, and community sports coaches. In each district, a consensus meeting (CM) was held with the local HSCPs. All participants had given informed consent. The aim of the CMs was to identify barriers and facilitators of the implementation of FPIs in the community, from the perspective of the HSCPs. During the CMs, the Practical, Robust, Implementation and Sustainability Model (PRISM)- framework was used (34, 35). This framework consists of 4 domains: *Intervention*, *Recipients*, *External Environment* and *Implementation and Sustainability infrastructure*. The PRISM framework was used since it is a comprehensive framework, allowing us to systematically identify important multilevel contextual factors (35). Also, PRISM was developed as a practical, actionable model, that both practitioners and researchers could use; therefore, it was suitable to use in the CMs in the current study (35).

At the start of the session, post-its were handed out to the HSCPs and they were asked to write down barriers and facilitators that influenced the implementation of fall prevention, from their perspective. Then, they placed the post-its into the most suitable PRISM domain on a working sheet. The CMs were conducted in separate meeting areas to ensure privacy. The sessions were facilitated by two researchers who acted as moderators. The CMs were not recorded due to pragmatic challenges that arise with recording focus group discussions (e.g., speaker identification). Also, recording of the CMs did not fit with the purpose of this study, which was to collect barriers and facilitators to implementation rather than to gain a deeper understanding of these determinants. One of the researchers wrote meeting notes of the sessions. Data from the working sheets and meeting notes were also analyzed following a directed qualitative content analysis approach, according to the constructs of CFIR (18). We chose to continue with the CFIR framework at this stage since the PRISM framework lacked clear definitions, guidance and measures to assist in understanding contextual determinants. Conversely, CFIR provides a taxonomy, codebook, and definitions of constructs to facilitate its applicability and usefulness (18, 36). Moreover, the CFIR is based on, among others, elements of the PRISM framework, both drawing on theories of behavior change and improvement science (18, 37), resulting in similar context dimensions across both frameworks (13) and allowing to transfer from PRISM to CFIR. During the last step of the analysis, comparisons between literature and CMs were made and results were combined per CFIR construct and domain.

## 3. Results

**Figure 2:**
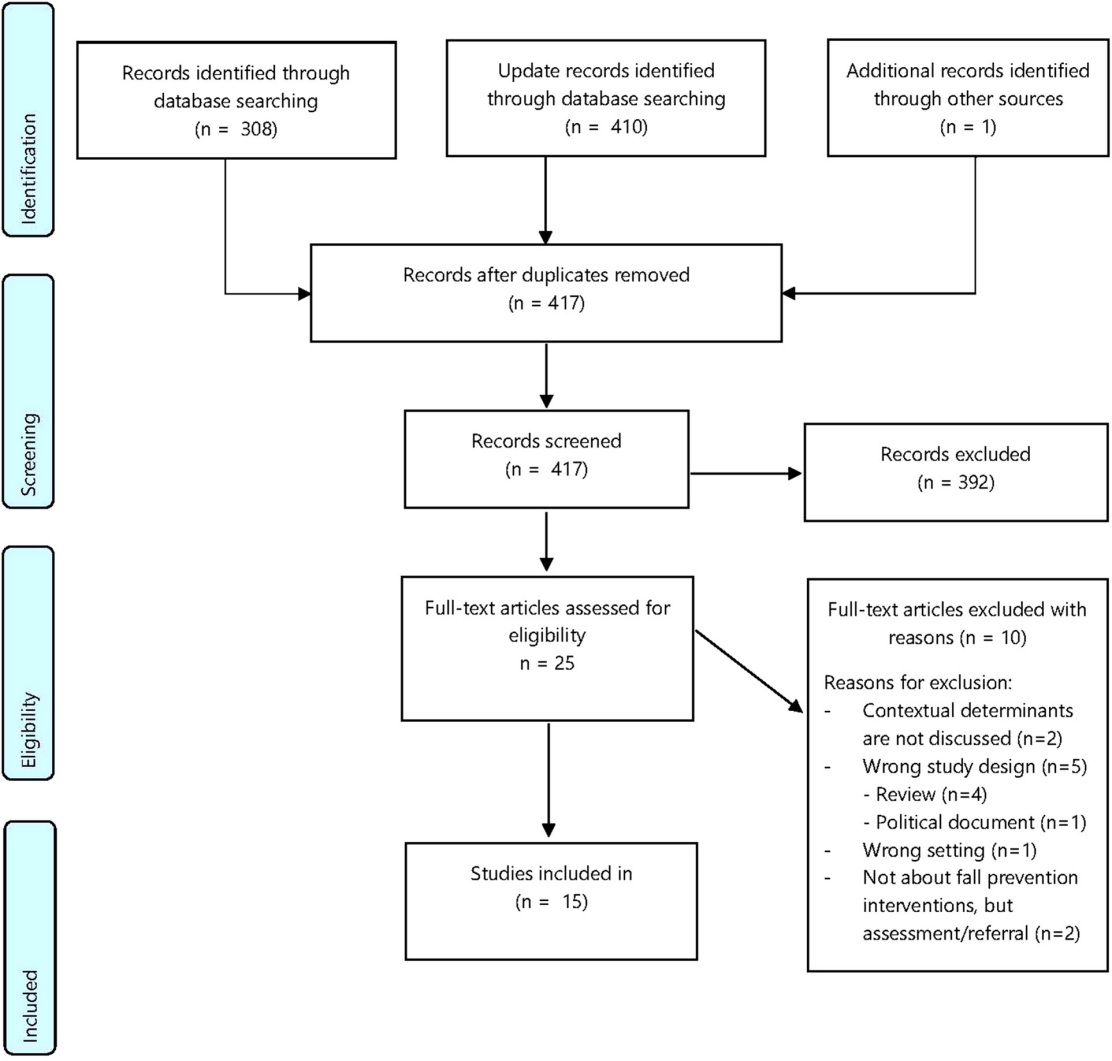
PRISMA-Flowchart regarding contextual determinants influencing the implementation of fall prevention interventions in a community setting.

The initial search strategy in electronic databases resulted in 308 studies; one additional study was added after screening through reference lists of the included studies. The updated search strategy in October 2022 and in March 2023 yielded 34 and 376 additional studies, respectively. Duplicates were removed (*n* = 302). A total of 392 studies were excluded after screening title and abstract, mainly because the implementation of fall prevention interventions was not discussed or the setting was not fitting, for example, studies on fracture prevention, implementation of person-environment approaches to prevent falls, the use of FPIs in hospitalized patients and integrated care for older adults in non-western countries. The remaining 25 articles were assessed fully for eligibility and 15 studies were finally selected for inclusion in this review (Figure 2) (38–52).

### 3.1. Descriptive data

#### 3.1.1. Literature

All included studies described barriers and facilitators to the implementation of FPIs in the community. Data-extraction of descriptive data showed that in seven studies (38–40, 43, 46, 48, 49), only health care professionals (e.g., physical therapists, general practitioners, pharmacists, allied health assistances, occupational therapists) were included. In the other studies, there was a combination of health care and social care professionals (e.g., informal care, social service workers) (41, 47) and/or other professionals (e.g., clinical support staff, fitness leaders, practice site leaders, volunteers) (42, 44, 45, 50–52). Furthermore, eleven studies (38–40, 42–45, 48–50, 52) focused primarily on primary care as setting; the other four studies (41, 46, 47, 51) concentrated on a broader community-based setting. Ten studies (39–41, 44–49, 52) used qualitative approaches only, such as semi-structured interviews and focus groups, whereas five studies (38, 42, 43, 50, 51) applied mixed methods with a qualitative and quantitative approach (e.g., surveys and documents). Of all included studies, three were conducted in Norway (39, 44, 45), three in Australia (40, 49, 51), three in the Netherlands (43, 48, 50), two in Canada (46, 47), two in Switzerland (38, 41) and two in the United States of America (42, 52) (Table 1).

**Table 1:** Descriptive data of the included studies.

Authors, year (country)	Study design	Data collection	Data analysis	Type of FPI	Setting	Relevant study population (sample size, gender, age—where specified in the study)
Amacher et al., 2016 (Switzerland)	Mixed methods design (exploratory sequential design)	Semi-structured interviews and postal surveys	Deductive content analysis	Multidisciplinary home-based fall prevention programme	Primary care	*N* = 12 GP [*n* = 4; female = 1; mean age = 55 (range 49–60)] HCN [*n* = 4; female = 4; mean age = 50 (range 48–54)] PT [*n* = 4; female = 4; mean age = 55 (range 49–59)]
Baumann et al., 2022 (Switzerland)	Qualitative design	Semi-structured focus group and semi-structured individual telephone interviews	Qualitative content analysis, based on Mayring	General fall prevention interventions	Community care, in two rural regions and one urban region	*N* = 28 Physician (GP, geriatric specialists, other specialists; *n* = 6) Occupational therapist (*n* = 7) Physical therapist (*n* = 6) Informal care (*n* = 4) Home care nurse (*n* = 3) Representative of senior's organization (*n* = 1)
Cerderbom et al.*,* 2020 (Norway)	Qualitative design (with a phenomenological perspective)	Semi-structured interviews	Content thematic analysis by Braun and Clark	General fall prevention interventions	Primary care (in municipalities (*n* = 6))	*N* = 17 PT; female = 11; age categories: 20–24 = 1, 25–29 = 1, 30–34 = 4, 35–39 = 5, 40–44 = 2, 45–49 = 2, 50–59 = 1, 60–69 = 1
Day et al.*,* 2016 (Australia)	Qualitative design	Semi-structured interviews	Deductive content analysis (according to the DOI attributes) and inductive analysis (of responses within the DOI attributes)	Group-based exercise fall prevention programs, based on the NoFalls Exercise Program, complemented with tai chi and/or group-based version of the OEP. Individual home-based exercise program based on the OEP	Primary care [PCPs in local government districts (*n* = 13)]	*N* = 17 PT (*n* = 14) OT (*n* = 1) AHA (*n* = 2)
Dykeman et al.*,* 2018 (Canada)	Qualitative descriptive research design	Semi-structured interviews and focus groups	Constant comparative method	Evidence-based fall prevention interventions	Public Health Units* in three geographical areas in Canada	*N* = 84 Female = 87%; median age = 50.5 (range 23–68)| HCP (*n* = 41); HCW (*n* = 10); administration (*n* = 10); other (*n* = 9); ESP (*n* = 4); recreation/fitness leader (*n* = 4); SSW (*n* = 4); retailer (*n* = 1); volunteer (*n* = 1)
Frazer et al.*,* 2021 (The Netherlands)	Quasi-experimental pre–post design (mixed methods)	Process evaluation (semi-structured interviews) and questionnaires	N/S	TOM	Community	*N* = 10 PTs (*n* = 4); dieticians (*n* = 2); volunteer (*n* = 2); local coordinator (*n* = 2)
Gemmeke et al.*,* 2022 (The Netherlands)	Implementation study, pre- post mixed methods design	Individual or double semi-structured interviews and registration data	Deductive analysis, using a topic list based on CFIR	Fall prevention service, consisting of 1) fall risk screening; 2) fall consultation to assess modifiable fall risk factors with accompanying interventions conducted by the pharmacy technician; 3) quick medication check and 4) comprehensive medication review if needed by the pharmacist.	Community pharmacies	*N* = 11 Pharmacists (*n* = 9; female = 5; mean age 44.4 ± 12) Pharmacy technicians (*n* = 1) Pharmacist-in-training (*n* = 1)
Johnston et al.*,* (2022) (United States of America)	Mixed methods design	Individual semi-structured interviews with key informants, intercept interviews with health care providers and clinical support staff, and fieldnotes and surveys	Qualitative content analysis, according the AIM domains of the RE-AIM framework	STEADI initiative	Non-profit health care organization, including primary care practices (*n* = 14)	*N* = 90 Key informants (*n* = 20): organizational administrators (*n* = 3); practice site leaders (*n* = 9); nurse leaders (*n* = 2); IT personnel (*n* = 3) Health care providers (*n* = 70): clinical support staff (*n* = 46); health care providers (*n* = 24)
Liddle et al.*,* 2018 (Australia)	Qualitative design	In-depth qualitative interviews	Thematic analysis	General fall prevention practices	Private and public sector setting	*N* = 15 Female (*n* = 13) PTs (*n* = 6), OTs (*n* = 4), EP (*n* = 2), podiatry (*n* = 3)
Markle-Reid et al.*,* 2017 (Canada)	Exploratory, retrospective multiple case study design	Semi-structured interviews, focus groups, documents (e.g. one-page story about the project and group's processes for working together)	Cross-case synthesis	General fall prevention interventions	Community	*N* = 32 PHPs divided over Ontario-based community groups (*n* = 4)
Meekes et al.*,* 2022 (The Netherlands)	Implementation study, qualitative design	Researcher's journal, i.e. unstructured informal interviews and online focus groups	Thematic analysis, supported by the Theoretical Domains Framework	Evidence-based fall prevention interventions: A Matter of Balance-NL, In Balance, Nijmegen Falls Prevention Program and OEP	Primary care	*N* = 11 Physiotherapists (*n* = 9), exercise therapists (*n* = 2)
Peel et al, 2010 (Australia)	Implementation trial design (mixed methods)	Daily activity logs, monthly status reports, falls planning days reports, mid-term and post-project key stakeholder semi-structured interviews	Synthesize, analyze, identify key themes	Evidence-based falls prevention interventions	HSDs (*n* = 12) divided over AHSs (*n* = 3)	*N* = 25 Key stakeholders with knowledge of fall prevention activities in their local area and representative from organizations involved in falls prevention
Reckrey et al, 2021 (United States of America)	Qualitative design	Individual and group semi-structured interviews	Deductive analysis using the CFIR framework as codebook and inductive analysis	STRIDE intervention	Primary care	*N* = 44 Falls Care Managers (*n* = 13) Research Staff (*n* = 23) Members of Central Project Management and the National Patient Stakeholder Council (*n* = 6)
Worum et al, 2019 (Norway) Worum et al, 2020 (Norway)**	Qualitative design, with phenomenological perspective	Focus groups and individual in-depth interviews	Thematic analysis by Braun and Clark	OEP	Geriatric department in a hospital and primary healthcare in the municipality	*N* = 12 PTs (female = 10; mean age = 43.4 (range 23–66) Hospital leader (*n* = 1); PT at a hospital (*n* = 1); specialist in-patient PT (*n* = 2); community health service leader (*n* = 1); PT in municipality (*n* = 4); specialist community PT (*n* = 2); section leader intermediate care (*n* = 1)

AIM, Adoption, Implementation, Maintenance; AHA, Allied Health Assistant; AHS, Area Health Services (public hospitals, residential aged care facilities, community sector); CFIR, Consolidated Framework for Implementation Research; DOI, diffusion of innovation; EP, exercise physiology; ESP, Emergency Services Provider; FPI, fall prevention intervention; FSO, Falls Safety Officer (to support and coordinate the implementation of fall prevention practices); GP, general practitioner; HCN, health care nurse; HCP, Health Care Professional; HCW, Health Care Worker; HSD, Health Service Districts; N/A, not applicable; N/S, not specified; OEP, Otago Exercise Programme; OT, occupational therapist; PCP, primary care partnerships (community health services, hospitals and local family medical practitioners in local coalition); PHP, Public Health Professional; PT, physical therapist; RE-AIM, Reach, Effectiveness, Adoption, Implementation, Maintenance; SSW, Social Services Worker; STEADI, Stopping Elderly Accidents, Deaths and Injuries; STRIDE, Strategies To Reduce Injuries and Develop confidence in Elders; TOM, [in Dutch] “Thuis Onbezorgd Mobiel”: a 14-week group-based FPP, including an exercise and nutrition component.

* Public Health Units are areas represented by Health Unit staff on different levels, such as business managers (management), nurses (regulated health professions), health promotors (other practitioners) and administrative assistants (administration).

** Second article from same authors, study was performed with the same population.

#### 3.1.2. Consensus meetings

In total, four CMs (a, b, c, and d) were held in four districts in the region of Utrecht, the Netherlands, with 35 HSCPs. All CMs lasted 120 min and there were on average 9 (range 7–13) HSCPs involved. Table 2 shows the descriptive data of the participants of the CMs.

**Table 2:** Descriptive data of the participants of the consensus meetings.

Community	Type of health or social care professional	Gender (%)
Community A	Total *n* = 7 Physical therapist, general practitioner, dietician, nursing specialist, community nurse (*n* = 2), occupational therapist	Female, 100%
Community B	Total *n* = 7 General practitioner, community nurse, occupational therapist (*n* = 2), audiologist, member community centre, community coordinator	Female, 57%
Community C	Total *n* = 8 Physical therapist, nurse practitioner, community nurse, pharmacist, community sport coach (*n* = 2), podiatrist, policy advisor	Female, 75%
Community D	Total *n* = 13 Physical therapist (*n* = 3), dietician, community nurse, occupational therapist, pharmacist, community coordinator, policy advisor, municipal employee (*n* = 2), member community center, social worker	Female, 92%

### 3.2. Analysis of the literature and consensus meetings

Directed qualitative content analysis of the included studies and the CMs yielded determinants operating as barriers and/or facilitators within 35 unique CFIR constructs; data from the CMs resulted in 21 unique constructs. All 21 constructs which were identified in data from the CMs were also found in the included studies; whereas the remaining 14 constructs were identified only in the included studies and not in the CMs. In most determinants, it was recognized that a facilitator (e.g., having enough time) became a barrier when there was a lack of it (e.g., lack of time). Consequently, most identified determinants can act both as barrier and facilitator. Also, it should be noted that, in some cases, the absence of a determinant was a facilitator (e.g., no complex intervention), whereas the absence of another was a barrier (e.g., intervention is not compatible). Analysis of the data from the literature and the CMs is categorized and discussed per CFIR-domain and -construct (Table 3).

**Table 3:** Analysis of identified barriers, facilitators or determinants without specific direction in the included studies and consensus meetings according to the domains and constructs of the CIFR framework.

**CFIR-DOMAINS and -CONSTRUCTS**	Amacher, et al. (2016)	Baumann, et al. 2022)	Cerderbom, et al. *(*2020)	Day, et al. (2016)	Dykeman, et al. (2018)	Frazer, et al. (2011)	Johnston, et al. (2022)	Gemmeke, et al. (2022)	Liddle, et al. (2018)	Markle-Reid, et al. (2017)	Meekes, et al. (2022)	Peel, et al. (2010)	Reckrey, et al. (2021)	Worum, et al. (2019)	Worum, et al. (2020)	CM A	CM B	CM C	CM D
**I. INTERVENTION CHARACTERISTICS**																			
Intervention source *Internal or external development of the intervention*																			
																		
																		
Evidence strength & Quality *Quality and validity of evidence*																			
																		
																		
Relative advantage *Advantage of intervention vs. alternative solution*																			
																		
																		
Adaptability *Ability of adapting, tailoring, refining the intervention*																			
																		
																		
Trialability *Ability to test the intervention on a small scale*																			
																		
																		
Complexity *Perceived difficulty of implementation*																			
																		
																		
Cost *Costs of the intervention and costs associated with implementation*																			
																		
																		
**II. OUTER SETTING**																			
Patient Needs & Resources *Patient needs are known and prioritized by the organization*																			
																		
																		
Cosmopolitanism *Organization is networked with external organizations*																			
																		
																		
Peer pressure *Competitive pressure to implement an intervention*																			
																		
																		
External Policy & Incentives *External strategies, including policy regulations, guidelines*																			
																		
																		
**III. INNER SETTING**																			
Structural Characteristics S*ocial architecture, age, maturity and size of an organization*																			
																		
																		
Networks & Communications *Nature and quality of webs of social networks and communications*																			
																		
																		
Culture *Norms, values and basic assumptions of an organization*																			
																		
																		
Implementation Climate *Absorptive capacity for change, rewarding for use of intervention*																			
																		
																		
- Tension for Change *Perception of an intolerable situation and needing to change*																			
																		
																		
- Compatibility *Alignment intervention with norms and fitting in workflows*																			
																		
																		
- Relative Priority *Perception of the importance of implementation*																			
																		
																		
- Organizational Incentives & Rewards *Extrinsic incentives, including promotions, raises in salary*																			
																		
																		
- Goals & Feedback *Goals are clearly communicated, acted upon and fed back to staff*																			
																		
																		
Readiness for Implementation *Tangible and immediate indicators of organizational commitment*																			
																		
																		
- Leadership Engagement *Commitment, involvement of managers and leaders*																			
																		
																		
- Available Resources *Including time, money, training, education, physical space*																			
																		
																		
- Access to Knowledge & Information *Ease of access to information and knowledge*																			
																		
																		
**IV. CHARACTERISTICS OF THE INDIVIDUALS**																			
Knowledge & Beliefs about the Intervention *Individuals’ attitudes towards and value placed on the intervention*																			
																		
																		
Self-efficacy *Individual belief in their own capabilities to achieve goals*																			
																		
																		
Other Personal Attributes *Other personal traits, including motivation, values, competence*																			
																		
																		
**V. PROCESS**																			
Planning *A scheme and tasks for implementation are developed*																			
																		
																		
Engaging *Attracting and involving appropriated individuals*																			
																		
																		
- Opinion Leaders *Individuals in an organization who have (in)formal influence*																			
																		
																		
- Formally Appointed (Internal) Implementation Leaders *Coordinator, project manager*																			
																		
																		
- Champions *Individuals who support and “drive through” an implementation*																			
																		
																		
- External Change Agents *External individuals who formally influence or facilitate decisions*																			
																		
																		
Executing *Carrying out or accomplishing implementation according to a plan*																			
																		
																		
Reflecting & Evaluating *Feedback about the progress and quality of implementation*																			
																		
																		

CFIR, Consolidated Framework for Implementation Research; CM, consensus meeting.

Legend: green, facilitator (+); red, barrier (−); blue, determinant without specific direction (+/−).

#### 3.2.1. Characteristics of the intervention

According to the results of seven studies (38, 42–44, 48, 49, 52) and two CMs^a,d^, a degree of “complexity” of the intervention influences implementation. In the study by Worum, et al. (2019) (44), participants highlighted that information on the intervention program is often perceived as complex, with terminological challenges and differently defined guidelines. This eventually leads to poorer use, and therefore unsuccessful implementation of an FPI. In both CMs^a,d^, the user-friendliness of guidelines was referred to as being important for successful implementation.

Furthermore, determinants were identified within the construct “relative advantage”. This construct is defined as the stakeholders' perception of the advantage of implementing the intervention vs. an alternative solution (18). This was identified in three studies (42, 43, 45) and two CMs^a,c^. In the studies by Gemmeke, et al. (2022) (43) and Johnston, et al. (2022) (42) it was recognized that health care professionals were motivated to implement the intervention since they were well aware that it contributed to decreased fall risk in older adults, which improved health outcomes and lower health care costs.

Determinants about the “cost” of the intervention derived from both literature (38, 42, 48) and three CMs^a,c,d^. This refers to the—sometimes significant—financial contribution which is required for participation in FPIs, which can be a major barrier to some older adults.

“Evidence strength & quality” of the intervention was identified more often in the literature than in the CMs (three studies (44, 45, 51) and one CM^d^, respectively). The construct “adaptability” was identified in as many CMs as studies [three studies (44, 45, 52) and three CMs^a,b,d^]. It seems to be important that interventions are tailored to the context where the implementation takes place (44).

Other determinants identified in the included studies only were within the constructs “intervention source” (42, 46) and “triability” (40, 45).

#### 3.2.2. Outer setting

“Patient needs and resources” was identified in ten studies (38–40, 42–45, 48, 49, 52) and mentioned in all CMs^a–d^. General practitioners in the study by Amacher et al. (2016) (38) experienced difficulty with recruiting seniors for FPIs, because of their reaction of “no need” or “refusal”. Health care professionals in the study by Liddle et al. (2018) (49) expressed that persuading older clients, who did not acknowledge they had a fall risk or that hazards needed to be addressed and that FPIs would be beneficial, was the most difficult part of their work regarding fall prevention. In the CMs, this was experienced as well by several participants: there was denial and a lot of resistance from clients regarding fall prevention.

In a total of twelve studies (38–42, 44–50) and all CMs it was mentioned that networking well with external organizations is required to successfully implement FPIs in the community. This is summarized by the construct “cosmopolitanism”. In the studies by Markle-Reid et al. (2017) (46) and Day et al. (2016) (40) it was stated that establishing connections with other organizations and community groups that are trying to achieve similar goals was essential. Consequently, strong connections are likely to enhance capacity through increased referrals (40, 48). Moreover, in the study by Dykeman et al. (2018) (47) participants stated that fall prevention requires a community-wide approach, where crossing organizational boundaries and inter-agency relationships were deemed necessary for optimal teamwork and successful fall prevention activities. Participants in the CMs mentioned that working together with many stakeholders in the community is challenging, since for them it is often unknown what kind of services other HSCPs deliver as part of FPIs, and how to reach and connect with each other on a regular basis.

Furthermore, the absence of accurate funding and policies could lead to a compromise of quality care (“External policy & incentives”) since is often inadequate to meet the demands of HSCPs and it makes the implementation of FPIs much more complex and less attractive. This issue was highlighted in eleven studies (38, 39, 41–45, 47–49, 52) and all CMs. In the study by Liddle et al. (2018) (49), health care professionals discussed that funding systems were often perceived as barriers since they are complicated to understand and constantly changing. In the study by Dykeman et al. (2018) (47), it was stated that legislation determines what kind of services could be provided for the client, and this was often restricting. Also, there is a need for clear guidelines for fall prevention, which professionals have to be familiar with (38, 44, 47). Participants in the CMs mentioned that health insurance companies and municipalities should be more clear about how HSCPs and seniors can be reimbursed for implementing and attending FPIs, respectively.

The use of friendly competition, i.e., “peer pressure”, was identified as a facilitator in the study of Johnston et al. (2022) (42).

#### 3.2.3. Inner setting

In a total of twelve studies (38–43, 45–49, 51) and all CMs, it was mentioned that having well-established and -working networks, with effective communication within an organization, was of utmost importance for successful implementation of FPIs (“Networks & communications”). For example, chaotic communication and not being open to others' perspectives were perceived barriers (45, 47). The importance of networks and communication was emphasized by the requirement of a multi-professional and multidisciplinary approach to fall prevention (38, 39, 43, 46, 49). According to the CMs, however, there is often a significant lack of collaboration, as every professional works solitarily from each other. In addition, a problem that arose from the CMs in this context was that there is usually no clarity on colleagues' roles and responsibilities while there often is an overlap in skills and experiences. This specific challenge emerged from the literature as well (47, 49).

Determinants within the construct “implementation climate-compatibility” were identified in five studies (40, 42–45) and three CMs^a–c^. In the study by Gemmeke et al. (2022) (43) it was highlighted that, to facilitate further implementation, integration of FPIs into regular interventions was preferred. In the CMs it was also discussed that it would be beneficial to integrate fall prevention within existing workflows regarding other chronic diseases, such as diabetes. However, this is currently often not the case. In addition, combining workflows between different organizations can be challenging.

Furthermore, limited time, staff capacity and financial resources, unavailable venues to provide the intervention, high staff turnover, lack of support, and inconsistency in staff education may hinder the possibility of concrete use of FPIs. This is summarized by the construct “readiness for implementation- available resources” and was identified in twelve studies (38–43, 45–49, 51) and all CMs^a–d^.

Determinants within the construct “readiness for implementation-leadership engagement” were identified more often in the CMs, compared to the included studies; in three CMs^a,c,d^, and one study (45), respectively. In the CMs, participants mentioned that they often felt a lack of support from key organizational leaders, such as the management team, which eventually did not allow them enough time to implement FPIs. In the study by Worum et al. (2020) (45), this was highlighted as an important facilitator: commitment enabled a clearer direction of the process and how to proceed.

“Readiness for implementation-access to knowledge & information” was identified in five studies (38, 42, 44, 47, 51); this was not mentioned in the CMs. General practitioners stated, in the study by Amacher et al. (2016) (38), that they needed adequate information and helpful documents to be able to participate well.

Other constructs within this domain that were identified in both literature and the CMs, were within the constructs “structural characteristics” (42, 52) “culture” (39, 45, 49)^;a,c,d^, “implementation climate-relative priority” (45, 48, 51)^;a,d^ (the perception of individuals within an organization that implementation of the new intervention is important), “implementation climate-organizational incentives & rewards” ^c^(42, 45, 49, 51). The constructs “implementation climate” (45, 49), “implementation climate-tension for change” (51) “implementation climate-goals and feedback” (45, 46) and “readiness for implementation” (45, 51) were only identified in the literature.

#### 3.2.4. Characteristics of the individuals

Determinants within the construct “knowledge & beliefs about the intervention” emerged in eleven studies (38–40, 43–50) and two CMs^a,c^. Negative beliefs of HSCPs, e.g., related to the nature of falls and effective measures, were at times a barrier to implementing FPIs (47). Also, in some cases, professionals were not aware of how the intervention must be performed, e.g., trying to recruit older adults for FPIs according to wrong selection criteria (38). On the other hand, professionals indicated that, as they executed the FPIs, they learned the benefits for both clients and themselves, which reinforced the importance of delivering FPIs in everyday practice (49, 50).

In ten studies (38, 39, 42–47, 49, 52) and three CMs^a,c,d^ it was identified that working with enthusiastic HSCPs, who are motivated, dedicated, optimistic, and passionate about prevention facilitates the implementation of FPIs. All these personal features are summarized by the construct “other personal attributes”. HSCPs should have the capability, competencies, skills, and experiences to implement FPIs successfully since they play a crucial role in preventing falls (39, 44, 46, 47). In the CMs, participants considered the level of enthusiasm and dedication as of utmost importance.

Furthermore, the individual belief in their capabilities to execute FPIs well, summarized by “self-efficacy”, was identified in only one study (43).

#### 3.2.5. Process

Evidence indicated that having a well-planned strategy, with clear directions for all involved stakeholders, is important regarding successful implementation. Determinants within this construct, “Planning”, were identified in six studies (41–43, 45, 48, 51) and three CMs^a,c,d^. Also, the development of a scheme or tasks in advance of the implementation endeavors might be helpful for successful implementation. This was highlighted in the study by Baumann et al. (2022) (41) where registration forms were developed to facilitate communication, which was considered useful by the participants in the study.

Furthermore, identifying and engaging the right stakeholders to establish partnerships helps to succeed the implementation process. Determinants within the construct, “engaging”, were identified in nine studies (39, 40, 42–47, 51) and two CMs^b,d^. According to participants in the studies of Peel et al. (2010) (51) and Markle-Reid et al. (2017) (46), a variety of stakeholders must be involved: clinicians, (public) health professionals, non-government organizations, and older people. In the CMs it became clear that it can be difficult to keep key stakeholders involved actively, hindering the accurate use of FPIs. Also, in some cases, the group of involved stakeholders is not complete, missing an HSCP with a crucial role in the implementation process (e.g., general practitioner).

Furthermore, a leader or coordinator of the implementation process is another important factor for the successful implementation of FPIs. Determinants within the construct “engaging- formally appointed internal implementation leaders” emerged in eight studies (39, 40, 42, 43, 45–47, 51) and all CMs^a–d^. In the study by Worum et al. (2020) (45) it was emphasized that implementation success could not be achieved without an active leader. Also, this leader should provide supportive and perseverant leadership, and it is their task to engage the entire organization and ensure that everyone is involved in and informed about the implementation process (45, 47). In the CMs, participants indicated an active leader is necessary to keep an overview of other projects in the community and keep the implementation process moving forward.

Several determinants were only identified in the literature and not in de CMs: constructs “engaging-opinion leaders” (45), “engaging- champions” (42, 47, 51), “engaging- external change agents” (45, 47, 49) and “executing” (41–43, 47).

Finally, “reflecting & evaluating” was identified in four studies (42, 45, 47, 51) and one CM^c^.

## 4. Discussion

The aim of this scoping review was to identify what contextual determinants influence the implementation of FPIs in the community. Although fall prevention requires a community-wide approach, where various stakeholders and organizations must cross boundaries, an overview of barriers and facilitators that influence implementation in this particular setting remained still unexplored. Directed qualitative content analysis of the literature and the four CMs identified determinants within all CFIR domains and in almost all (35 of the 39) CFIR constructs; suggesting that a broad array of barriers and facilitators influences the implementation of FPIs.

Also, all included studies and CMs reported multiple contextual determinants to implementation, emphasizing that successful implementation of FPIs in the community is challenging since there is not one single factor that can be identified as a key barrier or facilitator. This has been recognized in previous research as well (6, 53). However, findings in this review indicate that there are a few important determinants that definitely need to be considered when implementing an FPI in the community setting—since a relatively large overlap was shown between the identified determinants within the included studies and the CMs. One of these essential determinants is regarding working collaboratively with the right stakeholders, within and outside an organization. This collaboration theme was categorized under CFIR constructs such as “networks & communications” and “cosmopolitanism”, and was described in almost all included studies and mentioned in all CMs. In the CMs it was remarked that the unclarity of roles and responsibilities among involved stakeholders was often a challenge. These findings are in line with prior research, where strong cross-disciplinary and cross-organizational partnerships were identified as being of utmost importance which cannot be neglected in the scope of the multifactorial nature of fall prevention, where multiple stakeholders must be involved (6, 19, 23, 54). The recently published World Guidelines for Falls Prevention and Management for Older Adults also highlights that, for successful implementation, regular interaction and engagement with key stakeholders is required (3). Furthermore, appropriate leadership is important; strong project management and clear communication between leaders and implementers are needed to achieve successful implementation. Such leaders should be engaged in implementation activities to be successful (18). This has been found in previous research as well, both within the scope of fall prevention and in the broader view of evidence-based practice across health and social care settings (15, 55–57). Also, “available resources”, such as time, financing, and staff was identified frequently in the included studies and the CMs. This construct is categorized under the CFIR construct “readiness for implementation”, suggesting that when these aspects are taken care of, the readiness of an organization to implement a given intervention will increase (18). Other research has highlighted the importance of handling “available resources” during the implementation of evidence-based interventions, within the scope of fall prevention or other contexts, as well (19, 58–60). Finally, taking into account the wishes and needs of the patients appears to be of significant importance, such as practical issues (costs, transportation, location) and the usage of fall prevention-related language when reaching and interacting with older adults. Especially the latter has been shown as an essential aspect to consider, since older adults often do not recognize they have a fall risk that needs to be addressed, leading to reluctance to adhere to FPIs (61). Overall, it is possible that the abovementioned determinants act as core components that are less dependent on different contexts, and therefore always should be taken into account when implementing FPIs in a community setting.

In general, it should be highlighted that context matters in implementation practice; and this is emphasized by the results of this study. We found both a differentiation in the direction of identified determinants (i.e., barrier, facilitator, or having no specific direction) and a variety of identified CFIR constructs within and across included studies and involved communities. In detail, during the coding process in this study, a distinction was made between determinants that were explicitly mentioned as being a barrier or facilitator, and determinants without a specific direction. This resulted in a detailed overview, displaying that the majority of the identified determinants can act both as barriers and facilitators: a factor was a facilitator if it was present; its absence was considered a barrier. This has been acknowledged in other studies as well (58, 62), and could be due to the varying contexts where the implementation took place. Furthermore, we noticed that some constructs were only identified in the included studies and not in the CMs, while some constructs were identified more often in the CMs than in the included studies. This could also be due to the different contexts where the implementation occurred within the included studies and the involved communities. Nilsen et al. (2019) (13) stated that the specific context where the implementation of an evidence-based intervention is performed is considered responsible for study-to-study variations in outcomes. Hence, the results of this study can be used as an indication of which determinants might be important to consider while implementing FPIs, but the variation of identified determinants and constructs also underlines the importance of always taking into account local contexts (13, 63). The next step of the implementation process is to design tailored implementation strategies that specifically address previously identified determinants in its local context (10, 64).

There are several strengths to this review. First, gathering complementary data from stakeholders “in the field” has led to data representing determinants from a real-life setting. This allows us to, later on, select and design implementation strategies that fit to their local context, leading to the most effective results (13, 64). Also, consulting stakeholders in addition to the literature review has resulted in rich data; perspectives of both health and social care professionals are involved in this review. This is in line with recommendations from the current World Guidelines of Fall Prevention in Older Adults, which states that optimal implementation requires actions in healthcare and social care sectors (3). Second, the comprehensive and widely used CFIR was used, to ensure a systematical and clear approach to data analysis.

There are also a few limitations to this scoping review. The search strategy yielded studies that did not include older adults' perspectives. However, the description of the construct “patients” needs and resources' covers this issue partially. Also, in a sub-study of the FRIEND project, the views of older adults on fall prevention are studied more extensively. Results will be published in the near future, and therefore, we chose not to include this topic in the current review. Furthermore, different frameworks were used during the CMs and the analysis afterward. However, this may not have led to different results, since the raw data (i.e., the barriers and facilitators on the post-its) was used for further analysis. The reason for choosing the CFIR framework over PRISM to analyze data was that the CFIR provides a well-defined taxonomy that facilitates its usefulness as an explanatory framework to identify and understand the success or failure of implementation activities (18). PRISM lacks clear definitions and guidance to assist in planning, understanding, and improving results (37). Also, the alignment of the use of CFIR throughout the entire review allowed for not only, comparisons between findings in the literature and the CMs, but also for comparisons and building knowledge on what influences implementation across studies and contexts over time (63).

Unfortunately, an updated version of the CFIR was published after the analysis of the current review was completed and therefore, the older version (2009) was used. The updated CFIR expanded its number of determinants and other constructs were renamed, separated into multiple constructs or relocated to different domains (65). Despite many updates, the new constructs can still be mapped back to the original CFIR to ensure consistency over time (65). Besides, the constructs of the 2009-CFIR framework have been linked to a collection of implementation strategies that were developed by Powell et al. (2015) (66), helping to guide decisions about the strategies that match locally identified barriers (10). Therefore, the selected implementation strategies for the involved communities within the FRIEND project will fit the local context and, consequently, lead to better implementation outcomes. The constructs of the updated CFIR are not yet related to implementation strategies; this remains an area for future research.

Also, when tailored implementation strategies are applied, it is important to understand why a strategy did or did not reach the intended outcomes. Insight into working mechanisms of implementation strategies may help to inform determinant-strategy matching and eventually create a more rational compilation of strategies that target local determinants and, therefore, fit contextual challenges. Research on mechanisms has been started recently (67), but precise guidance and knowledge on this matter are still unknown and future implementation research on this topic should be performed (68).

In conclusion, to successfully move evidence into action, the first step is to understand the local context and the interplay between contextual determinants. Findings in the current review show that multiple determinants play a role in achieving successful implementation of FPIs in the community. However, establishing collaborative relationships, accounting for time, financing and staff, and appointing strong leaders seem to be of utmost importance to take into account, regardless of the context where the implementation occurs. Also, taking into account the wishes and needs of older adults while providing FPIs appears to be essential to successful implementation. Looking ahead, our task is now to select and design implementation strategies that fit the local context within the communities involved in this review, and to provide insight into the application and effectiveness of these strategies. This will eventually support a more widely and structurally applied implementation of FPIs, which ultimately reduces falls among our growing aging population.

## APPENDICES

**Table:** APPENDIX 1 PRISMA-ScR-Checklist (28).

SECTION	ITEM	PRISMA-ScR CHECKLIST ITEM	REPORTED ON PAGE #
**TITLE**			
Title	1	Identify the report as a scoping review.	1
**ABSTRACT**			
Structured summary	2	Provide a structured summary that includes (as applicable): background, objectives, eligibility criteria, sources of evidence, charting methods, results, and conclusions that relate to the review questions and objectives.	1
**INTRODUCTION**			
Rationale	3	Describe the rationale for the review in the context of what is already known. Explain why the review questions/objectives lend themselves to a scoping review approach.	2
Objectives	4	Provide an explicit statement of the questions and objectives being addressed with reference to their key elements (e.g., population or participants, concepts, and context) or other relevant key elements used to conceptualize the review questions and/or objectives.	2
**METHODS**			
Protocol and registration	5	Indicate whether a review protocol exists; state if and where it can be accessed (e.g., a Web address); and if available, provide registration information, including the registration number.	3
Eligibility criteria	6	Specify characteristics of the sources of evidence used as eligibility criteria (e.g., years considered, language, and publication status), and provide a rationale.	3
Information sources*	7	Describe all information sources in the search (e.g., databases with dates of coverage and contact with authors to identify additional sources), as well as the date the most recent search was executed.	3
Search	8	Present the full electronic search strategy for at least 1 database, including any limits used, such that it could be repeated.	3, 19
Selection of sources of evidence^†^	9	State the process for selecting sources of evidence (i.e., screening and eligibility) included in the scoping review.	3
Data charting process^‡^	10	Describe the methods of charting data from the included sources of evidence (e.g., calibrated forms or forms that have been tested by the team before their use, and whether data charting was done independently or in duplicate) and any processes for obtaining and confirming data from investigators.	3
Data items	11	List and define all variables for which data were sought and any assumptions and simplifications made.	3, 4
Critical appraisal of individual sources of evidence^§^	12	If done, provide a rationale for conducting a critical appraisal of included sources of evidence; describe the methods used and how this information was used in any data synthesis (if appropriate).	Not applicable
SECTION	ITEM	PRISMA-ScR CHECKLIST ITEM	REPORTED ON PAGE #
Synthesis of results	13	Describe the methods of handling and summarizing the data that were charted.	3–5
**RESULTS**			
Selection of sources of evidence	14	Give numbers of sources of evidence screened, assessed for eligibility, and included in the review, with reasons for exclusions at each stage, ideally using a flow diagram.	5
Characteristics of sources of evidence	15	For each source of evidence, present characteristics for which data were charted and provide the citations.	5
Critical appraisal within sources of evidence	16	If done, present data on critical appraisal of included sources of evidence (see item 12).	Not applicable
Results of individual sources of evidence	17	For each included source of evidence, present the relevant data that were charted that relate to the review questions and objectives.	5
Synthesis of results	18	Summarize and/or present the charting results as they relate to the review questions and objectives.	5–13
**DISCUSSION**			
Summary of evidence	19	Summarize the main results (including an overview of concepts, themes, and types of evidence available), link to the review questions and objectives, and consider the relevance to key groups.	13
Limitations	20	Discuss the limitations of the scoping review process.	14
Conclusions	21	Provide a general interpretation of the results with respect to the review questions and objectives, as well as potential implications and/or next steps.	15
**FUNDING**			
Funding	22	Describe sources of funding for the included sources of evidence, as well as sources of funding for the scoping review. Describe the role of the funders of the scoping review.	15

JBI, Joanna Briggs Institute; PRISMA-ScR, Preferred Reporting Items for Systematic reviews and Meta-Analyses extension for Scoping Reviews.

* Where *sources of evidence* (see second footnote) are compiled from, such as bibliographic databases, social media platforms, and Web sites.

^†^ A more inclusive/heterogeneous term used to account for the different types of evidence or data sources (e.g., quantitative and/or qualitative research, expert opinion, and policy documents) that may be eligible in a scoping review as opposed to only studies. This is not to be confused with *information sources* (see first footnote).

^‡^ The frameworks by Arksey and O’Malley (6) and Levac and colleagues (7) and the JBI guidance (4, 5) refer to the process of data extraction in a scoping review as data charting.

^§^ The process of systematically examining research evidence to assess its validity, results, and relevance before using it to inform a decision. This term is used for items 12 and 19 instead of “risk of bias” (which is more applicable to systematic reviews of interventions) to include and acknowledge the various sources of evidence that may be used in a scoping review (e.g., quantitative and/or qualitative research, expert opinion, and policy document).

**Table:** APPENDIX 2 Search strategy in Pubmed.

Domain	(“old” [Title/Abstract] OR “older adults” [Title/Abstract] OR “older population” [Title/Abstract] or elderly [Title/Abstract] or aging [Title/Abstract] or senior [Title/Abstract] or “older people” [Title/Abstract] or “aged 65” [Title/Abstract] or aging [Title/Abstract] or 65 + [Title/Abstract] OR “community-dwelling” [Title/Abstract] OR “Aged” [Mesh] OR “Aged, 80 and over” [Mesh])
	(“primary care” [Title/Abstract] OR “primary health care” [Title/Abstract] OR “community” [Title/Abstract] OR “community-based” [Title/Abstract] OR neighborhood [Title/Abstract] OR “health center” [Title/Abstract] OR “health services” [Title/Abstract] OR “residence” [Title/Abstract] OR “Residence Characteristics” [Mesh] OR “Community Medicine” [Mesh] OR “Community Health Services” [Mesh] OR “Community Health Centers” [Mesh] OR “Primary Health Care” [Mesh])
Determinant	(“Implementation” [Title/Abstract] OR “implementing” [Title/Abstract] OR “implement” [Title/Abstract] OR “implementation process” [Title/Abstract] OR “research translation” [Title/Abstract] OR “research-practice gap” [Title/Abstract] OR “Health Plan Implementation” [Mesh] OR “Implementation Science” [Mesh] OR “Diffusion of Innovation” [Mesh])
	(“falling” [Title/Abstract] OR “fall prevention” [Title/Abstract] or “fall prevention intervention” [Title/Abstract] OR “fall prevention program*” [Title/Abstract] OR “fall-prevention programmes” [Title/Abstract] OR “falls prevention” [Title/Abstract] OR “falls prevention intervention” [Title/Abstract] OR “falls prevention program*” [Title/Abstract] OR “preventing falls” [Title/Abstract] or “prevent falls” [Title/Abstract] or “fall intervention” [Title/Abstract] or “fall program” [Title/Abstract] OR “fall prevention practices” [Title/Abstract] OR “integrated care” [Title/Abstract] OR “Accidental Falls [MeSH]”)
Outcome	(“organizational factors” [Title/Abstract] OR “local factors” [Title/Abstract] OR implications [Title/Abstract] OR practice [Title/Abstract] OR influencing [Title/Abstract] OR factors [Title/Abstract] OR cultural [Title/Abstract] OR “cultural influences” [Title/Abstract] OR influences [Title/Abstract] OR “personal characteristics” [Title/Abstract] OR personal [Title/Abstract] OR “personal factors” [Title/Abstract] OR interpersonal [Title/Abstract] OR characteristics [Title/Abstract] OR “social factors” [Title/Abstract] OR behaviors [Title/Abstract] OR environmental [Title/Abstract] or “environmental factors” [Title/Abstract] OR Context [Title/Abstract] OR “Context factors” [Title/Abstract] OR “contextual issues” [Title/Abstract] OR “contextual factors” [Title/Abstract] OR “practical” [Title/Abstract] OR “practical issues” [Title/Abstract] OR “practical problems” [Title/Abstract] OR “implementation issues” [Title/Abstract] OR stakeholders [Title/Abstract] OR “stakeholders involvement” or “health practitioners” [Title/Abstract] OR barriers [Title/Abstract] OR facilitators [Title/Abstract] OR “facilities” [Title/Abstract]
NOT	NOT (mental [Title/Abstract])
Filters	Publication date: custom range (2010–2022) Language: English, Dutch
